# Do general practitioners and psychiatrists agree about defining cure from depression? The DEsCRIBE™ survey

**DOI:** 10.1186/1471-244X-11-169

**Published:** 2011-10-14

**Authors:** Koen Demyttenaere, Marc Ansseau, Eric Constant, Adelin Albert, Geert Van Gassen, Kees van Heeringen

**Affiliations:** 1 University Psychiatric Centre, Catholic University of Leuven, Campus Gasthuisberg, B-3000 Leuven, Belgium; 2 Department of Psychiatry and Medical Psychology, University and CHU of Liège, CHU Sart-Tilman (B35), B-4000, Liège, Belgium; 3 Department of Psychiatry, Catholic University of Louvain, Cliniques Universitaires Saint-Luc, Avenue Hippocrate 10, B-1200 Brussels, Belgium; 4 Department of Medical Informatics and Biostatistics, University of Liège, CHU Sart Tilman, B-4000 Liège, Belgium; 5 Medical Department, Lundbeck Belgium, Avenue Molièrelaan 225, B-1050 Brussels; 6 University Department of Psychiatry and Medical Psychology, Unit for Suicide Research, University of Ghent Hospital, De Pintelaan 185, B-9000 Ghent, Belgium

## Abstract

**Background:**

This study aimed to document the outcome dimensions that physicians see as important in defining cure from depression. The study also aimed to analyse physicians' attitudes about depression and to find out whether they affect their prescribing practices and/or the outcome dimensions that they view as important in defining cure.

**Methods:**

A 51-item questionnaire based on six validated scales was used to rate the importance of several depression outcome dimensions. Physicians' attitudes about depression were also assessed using the Depression Attitude Scale. Overall, 369 Belgian physicians (264 general practitioners [GPs]; 105 psychiatrists) participated in the DEsCRIBE^™ ^survey.

**Results:**

GPs and psychiatrists strongly agreed that functioning and depressive symptomatology were most important in defining cure; anxious and somatic symptomatology was least important. GPs and psychiatrists differed in their attitudes about depression (p *<*0.001). Logistic regression revealed that the attitudes of GPs - but not psychiatrists - were significantly associated with their rates of antidepressant prescription (p < 0.001) and that certain attitudes predicted which outcome dimensions were seen as important in defining cure.

**Conclusions:**

Belgian GPs and psychiatrists strongly agreed on which criteria were important in defining cure from depression but differed in their attitudes about depression. The outcome dimensions that were considered important in defining cure were influenced by physicians' attitudes - this was more pronounced in GPs than in psychiatrists.

## Background

In 2006, the US National Institute of Mental Health published an article regarding the possibility of finding a cure for mental disorders [1]. This paper was a call to health practitioners to set themselves more ambitious goals when treating patients with mental illness. Moreover, this initiative aimed to cause a paradigm shift in perceptions of depression and its cure. While such an effort is admirable the paper failed to take into account the complexity of mental disorders such as depression and the inadequacies in our current ability to monitor and define the disease. Depression is multifactorial, and its aetiology and presentation differ greatly from one patient to the next. Furthermore, decades of research have failed to find consistent biological markers for depression or for its outcomes. To date, clinical and sociodemographic characteristics, such as comorbid anxiety, age or employment status, are the most consistent prognostic factors in depression [2,3], while evaluation of outcomes in depression has been limited to changes in symptom severity and concepts such as response, remission, recovery, relapse and recurrence [4,5]. Remission is usually defined as a score of less or equal to 7 on the Hamilton Depression Rating Scale (17 item version) or less or equal to 10 on the Montgomery-Asberg Depression Rating Scale: remission is therefore defined only on the basis of absence of symptoms.

In reality, most patients with depression present with comorbid somatic symptoms [6] or anxiety often reaching a severity akin to that of an anxiety disorder or a somatoform disorder [7]. A recent study of patients with major depressive disorder has illustrated that these symptom clusters (pain, anxiety and depression) are important in patients' assessment of improvement [8]. Patients reportedly value symptom resolution, normalisation of function and quality of life (QoL) when assessing whether their depression has remitted [9,10].

Additional evidence suggests that current definitions of outcome are inadequate. For example, although many psychiatrists believe anhedonia to be a core symptom of depression, assessment of change during the treatment of depression is usually limited to a decrease in negative affect, while an increase in positive affect is neglected. Furthermore, although the Diagnostic and Statistical Manual of Mental Disorders (fourth edition) definition of major depressive disorder includes a functional criterion (i.e., symptoms cause clinically significant distress or impairment in social, occupational or other important areas of functioning), functioning is often neglected in the inclusion criteria and the outcome measures of randomised controlled trials (RCTs). In order to address the limitations of existing outcome measures, a number of published papers have begun to address the importance of QoL in quantifying patients' response to antidepressant treatment [11,12]. QoL potentially represents a generic, global outcome measure that allows comparison of treatment effects across different disorders.

While we assert that the current narrow outcome definitions in the treatment of depression present only the view of the medical field instead of the outcomes that matter most to patients, there is presently a paucity of published data on patients' views of what constitutes a good outcome in depression [13,10]. Moreover, the views of individual physicians on the different dimensions that are important in defining a good outcome are also poorly documented. Kerr and colleagues [14] have reported that the attitudes of general practitioners [GPs] and psychiatrists towards depression and the treatment of depression differ markedly and also determine their prescribing practice and treatment outcomes. It is therefore expected that the attitudes of GPs and psychiatrists are also likely to differ about how to define satisfactory outcomes in depression treatment.

PROact^® ^(PRognosis Optimisation by adequate customised therapy) is an initiative that aims to define cure from depression by creating a therapeutic contract between patients and physicians. This paper documents the first part of PROact^® ^- DEsCRIBE™ (DEfinition of the CRIteria of BEing cured). In DEsCRIBE™ physicians were asked to consider what they thought was important in defining cure in patients with depression. In addition, we analysed the attitudes of GPs and psychiatrists concerning depression and whether their attitudes affect their prescribing practices and/or predict the outcome dimensions that are ranked highest when defining cure from depression.

## Methods

### Study design

This study was conducted between March and August 2009. The Belgian Central Medical Database was used to select a random sample of GPs and psychiatrists. These physicians were contacted between March and July 2009 by randomly assigned Lundbeck Belgium sales representatives who asked about their willingness to participate in the study. Any physician who declined to participate was replaced with another physician drawn randomly from the Central Medical Database.

Physicians who agreed to participate were asked to complete a two-part, password-protected, electronic web-based questionnaire. Part I included questions regarding their demographic characteristics, clinical experience and a query about whether they prescribe antidepressants to ≤ 50% (low prescribers) or > 50% (high prescribers) of their patients with depression. Part I also required participants to complete the Depression Attitude Scale (DAS) [15] which consists of 20 statements regarding the physician's attitudes about depression and its treatment. Physicians were asked to rate their agreement with each statement of the DAS on a 5-point Likert scale (where 1 = strongly agree and 5 = strongly disagree).

Part II of the survey requested that physicians rate the importance of 51 items in determining whether a patient has been cured of depression (the DEsCRIBE**™ **questionnaire). Physicians rated each item on a 5-point Likert scale (where 1 = not important and 5 = very important). The six scales used in the DEsCRIBE**™ **questionnaire to measure depression, anxiety, somatic symptoms, positive affect, functional impairment and QoL were, respectively: the Patient Health Questionnaire-Depression subscale (PHQ-9; 9 items); the Hospital Anxiety and Depression Scale-Anxiety subscale (HADS-A; 7 items); the Patient Health Questionnaire-Somatic Symptoms subscale (PHQ-somatic; 13 items); the Positive And Negative Affect Schedule-Positive Affect subscale (PANAS-pos; 10 items); the Sheehan Disability Scale (SDS; 3 items); and the Abbreviated World Health Organization QoL scale (WHOQOL-BREF; 9 items).

### Statistical analysis

Results were summarised as mean and standard deviation (SD) for quantitative variables and scores; frequency tables were used for categorical findings. Mean values were compared by one-way analysis of variance and proportions were analysed by the chi-squared test. The 51 DEsCRIBE™ questionnaire items were ranked by decreasing mean score (i.e., by order of importance) both for the whole sample and for GPs and psychiatrists separately. Spearman's rank correlation coefficients were calculated to measure the association between the attitude statements. A factor analysis based on the maximum likelihood principle and with varimax rotation was applied separately to the attitude statements of GPs and psychiatrists. This method allows selecting the exact number of factors which explain at least 100% of the common variance. These factors were subsequently analysed with respect to the demographic features and prescribing behaviour of the physician (high vs. low prescribers).

The association between the attitude factors and the six DEsCRIBE™ outcome dimensions was also studied by multiple regression analysis. Logistic regression was used to assess the relationship between the prescribing pattern of the physician (high vs low) and the attitude factors. The association with each factor was expressed in terms of the odds ratio (OR) and its 95% confidence interval (CI). Results were considered statistically significant at the 5% level (p < 0.05). Calculations were performed using SAS (version 9.1 for Windows) and S-PLUS (version 6.1) statistical packages.

## Results

### Study population

In total, 1240 physicians were contacted, of whom 369 completed the survey (response rate of 30%). The characteristics of the physicians who completed the survey and of the general Belgian physician population are compared in Table 1. The mean age of the participating physicians and of the Belgian physician population were comparable, while the proportion of female physicians was 7-14% lower in the study population vs. the Belgian physician population.

**Table 1:** Characteristics of study participants versus the Belgian physician population^a^

	Survey population		Belgian physician population	
**Characteristic**	**Psychiatrists (n = 105)**	**GPs (n = 264)**	**Psychiatrists (n = 1611)**	**GPs (n = 14 888)**

Female (%)	36	22	43	36
Practice: private/institutional/both (%)	17/16/67	96/4/0	-	-
Mean age (years)	46	50	50	50
Mean duration of practice (years)	17	24	-	-
Number of patients with depression treated each month	71	33	-	-

GP, general practitioner.

^a^Based on physicians present in the Belgian Central Medical Database.

### Defining a patient who is cured of depression

Physicians were asked to rate the importance of each of the 51 items of the multidimensional DEsCRIBE™ questionnaire in defining cure. Among the PHQ-9 depressive symptoms, GPs and psychiatrists considered decreased interest, depressed mood and suicidal ideation to be most important in defining cure, whereas concentration, appetite and psychomotor retardation/agitation were considered to be least important (Table 2).

**Table 2:** Ranking of the nine items of the PHQ-9 depressive symptomatology outcome dimension in terms of their importance in defining cure from depression according to GPs and psychiatrists

Rank	Psychiatrists	Mean score	GPs	Mean score
1	Little interest or pleasure in doing things	4.52	Little interest or pleasure in doing things	4.39
2	Feeling down, depressed, or hopeless	4.43	Feeling down, depressed, or hopeless	4.25
3	Thoughts that you would be better off dead or of hurting yourself in some way	4.18	Thoughts that you would be better off dead or of hurting yourself in some way	4.07
4	Trouble falling or staying asleep, or sleeping too much	4.09	Feeling bad about yourself, or that you are failure, or have let yourself or your family down	4.00
5	Feeling tired or having little energy	4.03	Trouble falling or staying asleep, or sleeping too much	3.99
6	Feeling bad about yourself, or that you are failure, or have let yourself or your family down	3.96	Feeling tired or having little energy	3.93
7	Trouble concentrating on things	3.96	Trouble concentrating on things	3.67
8	Moving or speaking so slowly that other people could have noticed Or the opposite - being so fidgety or restless that you have been moving around a lot more than usual	3.66	Poor appetite or overeating	3.25
9	Poor appetite or overeating	3.54	Moving or speaking so slowly that other people could have noticed Or the opposite - being so fidgety or restless that you have been moving around a lot more than usual	3.17

GPs, general practitioner.

Physicians were asked to rank the importance of each item from 1 to 5, with 5 indicating greatest importance.

GPs and psychiatrists agreed strongly about the items that were most and least important in defining cure from depression (Table 3). The top 10 items for GPs and psychiatrists comprised 3 PHQ-9 depression items, 3 WHOQOL-BREF items, 2 SDS items and 2 PANAS-pos items. The 10 least important items were all PHQ-somatic items.

**Table 3:** Most and least important statements in defining cure in patients with depression

Psychiatrists		GPs	
**Item**	**Mean score**	**Item**	**Mean score**

**10 most important items in defining cure**			

Little interest or pleasure in doing things	4.52	Little interest or pleasure in doing things	4.39

*Occupational functioning*	*4.44*	*Feeling life is meaningful*	*4.37*

Social functioning/leisure	4.44	Social functioning/leisure	4.36

Being able to enjoy life	4.44	Being able to enjoy life	4.30

Feeling down, depressed or hopeless	4.43	Occupational functioning	4.27

Interested	4.33	Feeling down, depressed or hopeless	4.25

Feeling life is meaningful	4.32	Not feeling blue, depressed or anxious	4.13

Active	4.25	Interested	4.12

Thoughts that one would be better off dead	4.18	Active	4.09

Being able to concentrate	4.13	Thoughts that one would be better off dead	4.07

**10 least important items in defining cure**			

Stomach pain	2.66	Back pain	2.73

Dizziness	2.61	Dizziness	2.71

Back pain	2.58	Stomach pain	2.65

Pain in arms, legs or joints	2.47	Pain during intercourse	2.65

Shortness of breath	2.47	Shortness of breath	2.59

Constipation, loose bowels or diarrhoea	2.43	Pain in arms, legs or joints	2.48

Nausea, gas or indigestion	2.36	Nausea, gas or indigestion	2.48

Pain during intercourse	2.30	Fainting spells	2.31

Fainting spells	2.08	Constipation, loose bowels or diarrhoea	2.29

Menstrual pain	2.01	Menstrual pain	1.98

Physicians were asked to rank the importance of each item from 1 to 5, with 5 indicating greatest importance.

The 3 Diagnostic Statistical Manual of Mental Disorders criteria located in the top 10 are highlighted.

GP, general practitioner.

The ranking of the mean scores for the six scales was identical for GPs and psychiatrists, with the SDS and PHQ-9 depression scales considered the two most important scales in defining cure and the HADS-A and PHQ-somatic considered the two least important scales (Table 4).

**Table 4:** Importance of scales for assessing whether a patient has been cured of depression

	Psychiatrists		GPs	
**Scale**	**Rank**	**Mean score**	**Rank**	**Mean score**

SDS	1	4.23	1	4.13
PHQ-9	2	4.04	2	3.86
WHOQOL-BREF	3	3.86	3	3.84
PANAS-pos	4	3.79	4	3.72
HADS-A	5	3.34	5	3.22
PHQ-somatic	6	2.50	6	2.61

All physicians were asked to rank the importance of each item from 1 to 5, with 5 indicating greatest importance - the total mean score for all items from the scale was then calculated and used to rank the scales.

GP, general practitioner; HADS-A, Hospital Anxiety and Depression Scale-Anxiety subscale; PHQ-9, Patient Health Questionnaire-Depression Subscale; PHQ-somatic, Patient Health Questionnaire-Somatic Symptoms subscale; PANAS-pos, Positive And Negative Affect Schedule-Positive Affect subscale; SDS, Sheehan Disability Scale; WHOQOL-BREF, Abbreviated World Health Organization Quality of Life scale.

### Physicians' attitudes about depression

Highly significant differences (p ≤ 0.001) were found between the attitudes of GPs and psychiatrists for 10 of the 20 statements on the DAS (Table 5). Several relations were noted between some DAS statements. For example, "feeling comfortable treating patients with depression" (A9) and "finding the experience rewarding" (A15) were positively correlated in both groups of physicians (r = 0.33, p < 0.001 for GPs and r = 0.34, p < 0.001 for psychiatrists). It is interesting to note that GPs (but not psychiatrists) who are comfortable treating patients with depression (A9) and find it rewarding (A15) did not agree that an underlying biochemical abnormality is causative for severe cases of depression (A4) (r = -0.13, p < 0.05) and did not agree that psychotherapy tends to be unsuccessful in patients with depression (A16) (r = -0.13, p < 0.05). It is also interesting to note that psychiatrists (but not GPs) who agree that a nurse could be useful in supporting patients with depression (A11) disagree that depression reflects a characteristic response in patients that is not amenable to change (A10) (r = -0.29, p < 0.01).

**Table 5:** Depression Attitude Scale questionnaire results - only statements with a significant difference between GPs and psychiatrists are shown

Statement	Physicians who agreed with the statement (%)^a^	
	**Psychiatrists**	**GPs**

A1. Since starting my practice, I have seen an increase in the number of patients presenting with depressive symptoms	54	82***
A3. Most depressive disorders seen in general practice improve without medication	20	16**
A4. An underlying biochemical abnormality is the basis of severe cases of depression	86	73*
A5. It is difficult to differentiate whether patients are presenting with unhappiness or a clinical depressive disorder that needs treatment	11	29***
A8. Patients with depression are more likely to have experienced deprivation in early life than other people	54	37**
A9. I feel comfortable in dealing with the needs of patients with depression	87	55***
A10. Depression reflects a characteristic response in patients which is not amenable to change	2	7*
A12. The nurse could be a useful person to support patients with depression	87	53***
A13. Working with patients with depression is heavy going	46	68***
A14. There is little to be offered to those patients with depression who do not respond to treatment by GPs	10	23***
A15. It is rewarding looking after patients with depression	78	45***
A16. Psychotherapy tends to be unsuccessful in patients with depression	2	11**
A17. If patients with depression need antidepressants, they are better off with a psychiatrist than with a GP	54	3***
A18. Antidepressants usually produce a satisfactory result in the treatment of patients with depression in general practice	29	82***
A19. Psychotherapy for patients with depression should be left to a specialist	74	47***
A20. If psychotherapy was freely available, this would be more beneficial than antidepressants for most patients with depression	12	26**

GP, general practitioner.

*p ≤ 0.05; **p ≤ 0.01; ***p ≤ 0.001 for differences between the physician groups.

^a^Physicians who 'tended to agree' or 'strongly agree' with the statement on the Likert scale were compared to the others by the chi-square test.

A maximum likelihood (with varimax rotation) factor analysis of the DAS responses was performed separately for GPs and psychiatrists (Table 6). The analysis of GP responses revealed a three-factor solution. The factors were named after the statement with the highest loading (coefficient) within each factor:

**Table 6:** Factor analysis of Depression Attitude Scale statements in GPs and psychiatrists - only attitude statement with at least one loading ≥ 40 or ≤ -40 for any of the factors are represented

Statement	Factor 1		Factor 2		Factor 3		Factor 4	Factor 5
	**GP**	**Psych**	**GPs**	**Psych**	**GP**	**Psych**	**Psych**	**Psych**

A2	36	2	-11	22	13	14	-8	47*

A3	18	-5	-42*	-34	-26	4	-2	81*

A5	23	20	-45*	3	9	47*	1	6

A6	46*	25	2	21	3	-6	15	15

A7	70*	73*	-13	9	-8	-4	-24	16

A8	42*	28	8	26	-19	16	5	30

A9	8	-7	47*	56*	-25	-17	0	-10

A10	49*	36	-5	-10	29	40*	5	4

A14	20	-15	-19	8	44*	74*	8	-9

A15	-2	-6	48*	63*	-23	8	1	-3

A16	-3	-17	-7	-10	55*	50*	1	9

A17	15	16	-32	16	9	-3	85*	-1

A18	6	-1	40	12	7	-12	-55*	7

A19	18	-9	-17	40	23	-3	43*	12*

A20	30	22	-32	-17	-28	-17	6	44*

GP, general practitioner; Psych, psychiatrist.

*Statement loading ≥ 40 or ≤ -40.

Shading indicates highest scoring statements in each factor solution common between GP and psychiatrists (for factors 1 to 3). This statement was then used to name the solution as it is representative of an underlying theme in the attitudes of the physicians surveyed.

■ Factor 1: Depression is how people with poor stamina deal with stress.

■ Factor 2: It is rewarding looking after patients with depression.

■ Factor 3: Depression has a poor outcome.

By contrast, the analysis of psychiatrist responses revealed a five-factor solution. Three of these factors (Factors 1 to 3) were basically the same as those identified for GPs as the statements with the highest loading in Factors 1, 2 and 3 were the same for the psychiatrist and GP groups. The two additional factors for psychiatrists were:

■ Factor 4: Depression should be treated by psychiatrists.

■ Factor 5: Depression in primary care often resolves spontaneously (and needs psychotherapy rather than medication).

Comparison of the scores for the five factors between the two physician groups revealed that, compared with psychiatrists, GPs believe more strongly that depression is how people with poor stamina deal with stress (Factor 1; p < 0.0001) and that GPs feel it is less rewarding treating patients with depression (Factor 2; p < 0.0001). Moreover, GPs believe that there is little to offer to patients with depression who do not respond to treatment by GPs, but to a lesser extent than psychiatrists (Factor 3; p < 0.0001) and more strongly disagree that treating depression is the role of the psychiatrist (Factor 4; p < 0.0001). Compared with psychiatrists, GPs agree more with the statement that most depressive disorders in a primary care practice do not improve without medication and they disagree less with the statement that freely available psychotherapy would be more beneficial than antidepressants (Factor 5; p < 0.0001).

### Physician attitudes about depression and prescribing patterns

The present survey indicated that more psychiatrists than GPs are high prescribers of antidepressants - 69% of psychiatrists and 37% of GPs prescribed antidepressants to

> 50% of their patients with depression.

A logistic regression analysis with prescribing pattern (high vs. low prescribers) as the dependent variable and the three GP attitude factors and sociodemographic characteristics as independent variables revealed a significant association between GPs' perceptions and their prescribing behaviour (p *= *0.0067). Specifically, low prescribing was predicted by GPs' perception that looking after patients with depression is not rewarding (Factor 2, OR = 0.69; 95% CI: 0.49 to 0.97) and that depression has a poor outcome (Factor 3, OR = 0.62; 95% CI: 0.43 to 0.88).

A logistic regression analysis with prescribing pattern as the dependent variable and the psychiatrists' five depression attitude factors and sociodemographic characteristics as independent variables did not reveal any significant relationship between the depression attitude factors of psychiatrists and their prescribing behaviour (p = 0.068).

### Physician attitudes about depression and the importance of different outcome dimensions in defining cure

Six regression analyses were performed on the psychiatrist (and GP) data in order to assess whether the five (or three) depression attitude factors (in addition to gender and age) predicted the relative importance of each of the six outcome dimensions used in defining cure (ranking based on the mean score on each outcome dimension where 1 = lowest ranking and 6 = highest ranking).

A significant model was found for only two of the six outcome dimensions for psychiatrists. Psychiatrists who attach a greater relative importance to somatic symptomatology in defining cure agreed that it is rewarding looking after patients with depression (Factor 2; OR = 0.36; 95% CI: 0.16 to 0.82). Psychiatrists who attached a greater relative importance to functioning in defining cure agreed that depression is how people with poor stamina deal with stress (Factor 1; OR = 0.52; 95% CI: 0.33 to 0.81).

A significant model was found for five out of the six outcome dimensions for GPs. GPs who attach a greater relative importance to depressive symptomatology in defining cure agreed more strongly that depression is how people with poor stamina deal with stress in their lives (Factor 1; OR = 0.64; 95% CI: 0.49 to 0.84). GPs who think that anxious symptomatology is important in defining cure disagreed more strongly that depression is how people with poor stamina deal with stress in their lives (Factor 1; OR = 1.31; 95% CI: 1.00 to 1.71) and disagreed more strongly that depression has a poor outcome (Factor 3; OR = 1.40; 95% CI: 1.04 to 1.89). GPs who attach a greater relative importance to functioning in defining cure agreed more strongly that depression is how people with poor stamina deal with stress in their lives (Factor 1; OR = 0.62; 95% CI: 0.47 to 0.82) and disagreed more strongly that depression has a poor outcome (Factor 3; OR = 1.36; 95% CI: 1.00 to 1.84). GPs who think that positive affect is important in defining cure disagreed more strongly that depression is how people with poor stamina deal with stress in their lives (Factor 1; OR = 1.77; 95% CI: 1.35 to 2.32). GPs who attach a greater relative importance to QoL in defining cure were more likely to be women (OR = 1.99; 95% CI: 1.10 to 3.61) and agreed more strongly that depression has a poor outcome (Factor 3; OR = 0.62; 95% CI: 0.46 to 0.84).

## Discussion

The primary study finding was that GPs and psychiatrists give very similar responses when asked about which depressive symptoms and broader outcome dimensions are most important in defining cure from depression. Indeed, when the nine depression items of the PHQ-9 were ranked by these physician groups, the three most and least important items were identical. To the best of our knowledge this is the first documented comparison of GPs' and psychiatrists' opinions about defining cure from depression.

The 10 most important items in defining cure comprised the PHQ-9 items of anhedonia, depressed mood and suicidal ideation, the SDS items of occupational functioning and social functioning, the WHO-QOL items of being able to enjoy life, being able to concentrate and feeling life is meaningful and the PANAS-pos items of being interested and being active. The 10 least important items were all items from the somatic symptom scale. This is a remarkable finding as the importance of painful and non-painful symptoms has recently received a great deal of attention in the scientific literature [2,7,16,17].

Ranking the outcomes at the dimension level gives a broader definition of what a useful outcome in clinical practice might be. This contrasts with the narrow outcome definitions espoused by RCTs, which tend to focus on depression scale scores, response and remission [18]. It has been documented that patients enrolled in RCTs rarely typify the patients seen in clinical practice [19], and our data illustrate that the concept of cure encompasses more than can be captured by single scale scores and the current notions of response and remission. This idea has been mooted previously in the published literature [4,20] and has been stated particularly elegantly by Linsey McGoey of the Said Business School, University of Oxford: "Never before have the inadequacies of RCTs been so apparent to so many. Yet equally, never before have those in positions of authority - from regulators, to NICE policy makers, to doctors - relied so extensively on RCT evidence" [21]. Our data are indeed an illustration of the previous statement since endpoints in RCTs and endpoints in physicians' view seem to be very different.

The broader definition of cure from depression championed here seems to agree largely with published data reporting what patients consider to be important in defining cure. Patients give highest priority to positive mental health (optimism, vigour and self-confidence) followed by feeling normal, a return to usual levels of functioning at work or at home, feeling in emotional control, participating in and enjoying relationships with family and friends and, finally, the absence of depressive symptoms [9].

Analysis of these broader outcome dimensions is also instructive when looking at the concepts of relapse and recurrence. Residual deficits after treatment should not refer only to residual symptoms, rather they should encompass impairments in social and occupational functioning (even independently of depressive symptom scores). These broader outcome dimensions have been reported to be significant and independent risk factors for relapse and recurrence [22-24].

A second finding of this study were the important differences in attitudes about depression and its treatment demonstrated by GPs and psychiatrists (as measured using the DAS). Overall, psychiatrists have a more positive attitude towards depression and its treatment - this has also been reported in a study based in Wales [14]. In the current study, a factor analysis gave a different solution for GPs (3 factors) and psychiatrists (5 factors; 3 of them being the same as for the GPs). A 4-factor solution was reported in a study of 72 GPs by Botega and colleagues [15], where 3 of the 4 factors are mainly comparable to 3 of the 5 factors reported here. Factors I (antidepressant/psychotherapy), II (professional unease) and III (inevitable course of depression) in the Botega paper [15] correspond with Factors 2, 3 and 5 in our sample. It is a matter of concern that GPs feel that treating patients with depression is unrewarding (Factor 2) but it is interesting that GPs are pessimistic about what to do if a patient does not respond to their treatment (Factor 3) and that they feel most of their depressed patients should be treated with antidepressants (Factor 5).

The importance of physicians' attitudes to depression and their ability to manage this disorder effectively have been commented upon previously [14,15,25]. For example, one study [25] used a modified form of the DAS and found that non-psychiatrist physicians in Taiwan who were positive about the treatment of depression did not display avoidant/helpless attitudes and had the best scores in depression management. These findings support an earlier study of GPs in Scotland that also used the DAS [26]. This study reported that pessimism associated with the treatment of depression was linked to unwillingness to become involved with managing patients with depression, while confidence resulted in earlier recognition of the disorder [26]. Feeling comfortable with treating depression was also linked to more accurate diagnosis in a study of GPs in the north of England [27]. This study concluded that the accurate identification and appropriate management of depression by GPs was not an independent variable; instead it differed with different physicians' attitudes and skills [27].

It is perhaps unsurprising that psychiatrists are more comfortable treating patients with depression and find the experience more rewarding than GPs as they are specialists in this field. It has been reported that mental health expertise among GPs is also helpful in improving their attitudes towards the treatment of depression. An earlier study of GPs using the DAS revealed that physicians who had gained postgraduate mental health qualifications were more optimistic about achieving positive outcomes for their patients with depression and felt more comfortable in assisting these patients [28]. By contrast, a study of primary care physicians' attitudes to depression conducted in Brazil illustrates that a lack of exposure to patients with depression has the opposite effect [29]. The results of such surveys are useful as they suggest that training and experience can influence physicians' comfort in dealing with depression. It should also be considered that attitudes may influence participation in training programmes and influence the patients that physicians treat within their own practice. The finding that psychiatrists' attitudes do not appear to influence how they treat their patients is harder to explain. Perhaps the greater experience of psychiatrists concerning mental illness leads them to be less influenced by personal attitudes.

A third finding of this study is that compared with psychiatrists, GPs prescribe antidepressants to a much smaller proportion of their patients with depression. This could of course be due to lower severity of depression in the patients seen by GPs and/or by the fact that in Belgium most patients first seek help for depression in primary care and are often only treated by a specialist when a first-line modality has failed. However, our data suggest that prescribing patterns are also predicted by physicians' attitudes, although this was only the case for GPs, not psychiatrists. To the best of our knowledge, this is a novel finding that has not been previously reported. Our study also indicates that low prescribing in GPs (but not in psychiatrists) is predicted by the following attitudes: that it is not rewarding to look after patients with depression (Factor 2) and that depression has a poor outcome (Factor 3). Studies regarding the relationship between physicians' attitudes and their prescribing patterns are scarce. Botega and colleagues [15] identified a subgroup (n = 26/72) of high-prescribing GPs. Analysis of low-dose prescribers among GPs in a study in Wales [14] indicated that, compared with standard-dose prescribers, this group were more in favour of psychotherapy as a treatment modality, agreed less strongly that depression has a biological basis, and agreed less strongly that depression could be treated effectively with antidepressants. Dowrick and colleagues [27] found that GPs' attitudes (measured using the DAS) did not predict the frequency with which they prescribed antidepressants; however, more positive attitudes regarding the biological basis of depression did predict the class of antidepressants that they prescribed (selective serotonin reuptake inhibitors). The present data suggest that, for GPs at least, finding it rewarding to look after patients with depression is correlated with more positive attitudes towards psychotherapy and with a less strong belief in a biological basis for depression. One could therefore speculate that these physicians find a good doctor-patient relationship more satisfying than they do prescribing an antidepressant.

A final finding of our study was that the outcome dimensions considered to be important in defining cure from depression were associated with physicians' attitudes towards depression. This result was more pronounced in GPs than in psychiatrists. It is difficult to interpret this finding, especially as overall both physician groups agreed strongly on the outcome dimensions that were important in defining cure. Our perception is that this result is consistent with the earlier finding that GPs are more influenced by their attitudes about depression than are psychiatrists. The data also suggest female GPs attach more importance to QoL issues than their male colleagues.

As with any survey, the findings reported here are only valid for the physicians who participated. The characteristics of physicians who declined to participate were not described and comparison of basic demographic characteristics revealed that the survey population consisted of a smaller percentage of women than the general Belgian physician population. However, refusal to participate is a universal issue in observational studies and on this issue our study was no more or less biased than any other study of similar methodology.

Our study asked physicians to rate whether they prescribe antidepressants to ≤ 50% or > 50% of their patients with depression. Responses to this question could be subject to recall bias and may be influenced by physicians' attitudes. Furthermore, the types of patients that are seen by psychiatrists and GPs differ. In Belgium, patients with depression are initially treated by a GP who then refers patients requiring second-line treatment to a psychiatrist. Consequently, GPs may treat more patients with depressive adjustment disorder while psychiatrists see more patients with major depressive disorder. The attitudes, beliefs and treatment patterns of GPs and psychiatrists are therefore likely to vary in response to the differences in their respective patient populations.

## Conclusions

The present study illustrates that (Belgian) GPs and psychiatrists strongly agree on the criteria that are important in defining cure from depression but strongly differ in their attitudes towards depression. Psychiatrists prescribe antidepressants to a larger proportion of their patients with depression compared with GPs. Prescribing patterns are significantly influenced by physicians' attitudes about depression, but only in GPs. The outcome dimensions considered to be most important in defining cure are also influenced by physicians' attitudes - this finding was more pronounced for GPs than for psychiatrists. Future research should address patients' perceptions of what defines cure and whether these attitudes correspond with those of their physician. Such research could even assess whether convergence or divergence between physicians' and patients' expectations about cure influences outcome or treatment satisfaction.

## Competing interests

KD has served as a consultant for AstraZeneca, Boehringer Ingelheim, Bristol-Myers Squibb, Eli Lilly, GlaxoSmithKline, Lundbeck, Servier, Takeda and Wyeth; EC has served as a consultant for AstraZeneca, Eli Lilly and Servier and has served as a member of speaker bureaus for AstraZeneca, Eli Lilly, Bristol-Myers Squibb, Lundbeck and Janssen. AA, MA and KvH have no financial or non-financial interests that may be relevant to the submitted work. GVG is a full-time employee of Lundbeck Belgium.

## Authors' contributions

All authors were involved in the analysis and interpretation of the data in this manuscript and were involved in the drafting and revising of the manuscript for intellectual content. All authors read and approved the final manuscript.

## Pre-publication history

The pre-publication history for this paper can be accessed here:

http://www.biomedcentral.com/1471-244X/11/169/prepub
